# Supplementary material for: Strategies for spreading, scaling and sustaining perinatal mental health interventions in low- and middle-income countries (LMICs): A scoping review and thematic synthesis
Source: Glob Ment Health (Camb). 2026 Apr 7;13:e96. doi: 10.1017/gmh.2026.10198 (PMC13184658; doi:10.1017/gmh.2026.10198)
Supplement: Sanfilippo et al. supplementary material [file S2054425126101988sup001.docx]

**Supplementary Materials**

**Strategies for spreading, scaling, and sustaining perinatal mental health interventions in Low- and Middle-Income Countries (LMICs) – A scoping review and thematic synthesis**

**Table of contents**

Supplementary Material 1. PRISMA-ScR checklist 2

Supplementary Material 2. Grey literature search 4

Supplementary Material 3. Search strategy for MedLine  6

Supplementary Material 4. Extraction table 7

**Supplementary Material 1. Preferred Reporting Items for Systematic reviews and Meta-Analyses extension for Scoping Reviews (PRISMA-ScR) Checklist**

| **SECTION** | **ITEM** | **PRISMA-ScR CHECKLIST ITEM** | **REPORTED ON PAGE #** |
| --- | --- | --- | --- |
| **TITLE** | | | |
| Title | 1 | Identify the report as a scoping review. | ​​Page 1​ |
| **ABSTRACT** | | | |
| Structured summary | 2 | Provide a structured summary that includes (as applicable): background, objectives, eligibility criteria, sources of evidence, charting methods, results, and conclusions that relate to the review questions and objectives. | ​​Page 2​ |
| **INTRODUCTION** | | | |
| Rationale | 3 | Describe the rationale for the review in the context of what is already known. Explain why the review questions/objectives lend themselves to a scoping review approach. | ​​Page 3-4​ |
| Objectives | 4 | Provide an explicit statement of the questions and objectives being addressed with reference to their key elements (e.g., population or participants, concepts, and context) or other relevant key elements used to conceptualize the review questions and/or objectives. | ​​Page 3-4​ |
| **METHODS** | | | |
| Protocol and registration | 5 | Indicate whether a review protocol exists; state if and where it can be accessed (e.g., a Web address); and if available, provide registration information, including the registration number. | ​​Page 4​ |
| Eligibility criteria | 6 | Specify characteristics of the sources of evidence used as eligibility criteria (e.g., years considered, language, and publication status), and provide a rationale. | ​​Page 4 and Table 1​ |
| Information sources* | 7 | Describe all information sources in the search (e.g., databases with dates of coverage and contact with authors to identify additional sources), as well as the date the most recent search was executed. | ​​Page 4​ |
| Search | 8 | Present the full electronic search strategy for at least 1 database, including any limits used, such that it could be repeated. | ​​Suppl Material​ |
| Selection of sources of evidence† | 9 | State the process for selecting sources of evidence (i.e., screening and eligibility) included in the scoping review. | ​​Page 4​ |
| Data charting process‡ | 10 | Describe the methods of charting data from the included sources of evidence (e.g., calibrated forms or forms that have been tested by the team before their use, and whether data charting was done independently or in duplicate) and any processes for obtaining and confirming data from investigators. | ​​Page 4​ |
| Data items | 11 | List and define all variables for which data were sought and any assumptions and simplifications made. | ​​Page 4​ |
| Critical appraisal of individual sources of evidence§ | 12 | If done, provide a rationale for conducting a critical appraisal of included sources of evidence; describe the methods used and how this information was used in any data synthesis (if appropriate). | ​​NA​ |
| Synthesis of results | 13 | Describe the methods of handling and summarizing the data that were charted. | ​​5​ |
| **RESULTS** | | | |
| Selection of sources of evidence | 14 | Give numbers of sources of evidence screened, assessed for eligibility, and included in the review, with reasons for exclusions at each stage, ideally using a flow diagram. | ​​Figure 1​ |
| Characteristics of sources of evidence | 15 | For each source of evidence, present characteristics for which data were charted and provide the citations. | ​​Table 2, Figure 2 and Page 5​ |
| Critical appraisal within sources of evidence | 16 | If done, present data on critical appraisal of included sources of evidence (see item 12). | ​​NA​ |
| Results of individual sources of evidence | 17 | For each included source of evidence, present the relevant data that were charted that relate to the review questions and objectives. | ​​Page 5​ |
| Synthesis of results | 18 | Summarize and/or present the charting results as they relate to the review questions and objectives. | ​​Page 5-9​ |
| **DISCUSSION** | | | |
| Summary of evidence | 19 | Summarize the main results (including an overview of concepts, themes, and types of evidence available), link to the review questions and objectives, and consider the relevance to key groups. | Page 9-10 |
| Limitations | 20 | Discuss the limitations of the scoping review process. | ​​Page 10​ |
| Conclusions | 21 | Provide a general interpretation of the results with respect to the review questions and objectives, as well as potential implications and/or next steps. | ​​Page 10-11​ |
| **FUNDING** | | | |
| Funding | 22 | Describe sources of funding for the included sources of evidence, as well as sources of funding for the scoping review. Describe the role of the funders of the scoping review. | ​​Page 5 and Page 11​ |

JBI = Joanna Briggs Institute; PRISMA-ScR = Preferred Reporting Items for Systematic reviews and Meta-Analyses extension for Scoping Reviews.

* Where *sources of evidence* (see second footnote) are compiled from, such as bibliographic databases, social media platforms, and Web sites.

† A more inclusive/heterogeneous term used to account for the different types of evidence or data sources (e.g., quantitative and/or qualitative research, expert opinion, and policy documents) that may be eligible in a scoping review as opposed to only studies. This is not to be confused with *information sources* (see first footnote).

‡ The frameworks by Arksey and O’Malley (6) and Levac and colleagues (7) and the JBI guidance (4, 5) refer to the process of data extraction in a scoping review as data charting*.*

§ The process of systematically examining research evidence to assess its validity, results, and relevance before using it to inform a decision. This term is used for items 12 and 19 instead of "risk of bias" (which is more applicable to systematic reviews of interventions) to include and acknowledge the various sources of evidence that may be used in a scoping review (e.g., quantitative and/or qualitative research, expert opinion, and policy document).

*From:* Tricco AC, Lillie E, Zarin W, O'Brien KK, Colquhoun H, Levac D, et al. PRISMA Extension for Scoping Reviews (PRISMAScR): Checklist and Explanation. Ann Intern Med. 2018;169:467–473. [doi: 10.7326/M18-0850](http://annals.org/aim/fullarticle/2700389/prisma-extension-scoping-reviews-prisma-scr-checklist-explanation).

**Supplementary Material 2. Grey literature search using the websites of organisations that focus on perinatal mental health**

| **Organisation** | **Resources** |
| --- | --- |
| Mental Health Innovation Network | <https://www.mhinnovation.net/innovations/thinking-healthy-pilot-peru?mode=wyocyiyvuws>  <https://www.mhinnovation.net/innovations/perinatal-mental-health-project?mode=hpidmhxjuseb> |
| Global Alliance for Maternal Mental Health | <https://globalalliancematernalmentalhealth.org/wp-content/uploads/2017/06/ks31.pdf>  <https://globalalliancematernalmentalhealth.org/wp-content/uploads/2017/06/Maternal-Mental-Health_Service-Guideline_PMHP.pdf> |
| The International Marcé Society for Perinatal Mental Health | <https://link.springer.com/article/10.1007/s00737-021-01160-1> |
| World Bank | <https://www.worldbank.org/en/what-we-do> |
| Gates foundation | <https://www.ncbi.nlm.nih.gov/pmc/articles/PMC7936480/>  <https://www.gatesgenderequalitytoolbox.org/mnch/> |
| Design in Mental Health | <https://www.designinmh.com/catalyst> |
| Clinton foundation | <https://www.clintonfoundation.org/>  <https://www.clintonfoundation.org/commitment/clinton-global-initiative/community-based-maternal-mental-health-care-in-uganda/> |
| United Nations | General Search on Google Search Engine |
| Médecins Sans Frontières | General Search on Google Search Engine |
| National Institutes of Health | <https://www.nimh.nih.gov/about/organization/cgmhr/scaleuphubs/nimh-scale-up-hubs-project-summaries> |
| NIHR | <https://www.nihr.ac.uk/news/research-funding-boost-for-mental-health-in-low-and-middle-income-countries/24900> |
| UNICEF | General Search on Google Search Engine |
| UK Aid ID | <https://drive.google.com/file/d/1uVPX7rHzWFdJo3L66__2CgddEknqRDPo/view> |
|  | <http://www.prime.uct.ac.za/implementation_phase_publications> |
| Australian Aid | General Search on Google Search Engine |
| Wellcome | General Search on Google Search Engine |
| Rockefeller foundation | General Search on Google Search Engine |
| MRC/UKRI | General Search on Google Search Engine |
| USAID/ MOMENTUM | <https://usaidmomentum.org/wp-content/uploads/2021/09/GECO-357_MCGL-CMPD-Landscape-Analysis_12-21-2021_Sec.508comp_v1.pdf>  <https://www.newsecuritybeat.org/2022/10/crisis-perinatal-mental-health-requires-collaborative-solutions/> |
|  | <https://www.alignmnh.org/mmh-evidence/> |
| WHO | <https://www.who.int/publications/i/item/9789240057142>  <https://www.who.int/publications/i/item/WHO-RHR-09.24> |
| PAHO | <https://docs.bvsalud.org/biblioref/2021/08/912908/who-recommendations-on-health-promotion-interventions-for-mater_St9J2il.pdf>  <https://pesquisa.bvsalud.org/global-literature-on-novel-coronavirus-2019-ncov/resource/pt/covidwho-2273338>  <https://www.paho.org/en/news/4-9-2021-first-mhgap-and-maternal-and-child-health-training-indigenous-health-care-workers> |
| African Alliance for Maternal Mental Health (AAMMH) | General Search on Google Search Engine |
| Global Alliance of Maternal Mental Health | \| <https://journals.plos.org/plosmedicine/article?id=10.1371/journal.pmed.1002385> \| \| --- \| \| [10.2196/jmir.6712](https://doi.org/10.2196/jmir.6712) \| \| <https://www.sciencedirect.com/science/article/pii/B978032391709400010X#bib35> \| \| <https://www.who.int/initiatives/mental-health-action-plan-2013-2030> \| |
| AlignMNH |  |
| TPO (Global NGO - Nepal and Uganda) | General Search on Google Search Engine |
| MacArther Foundation | General Search on Google Search Engine |

**Supplementary Material 3. Search strategy for MedLine**

Perinatal terms

1. (perinatal OR antenatal OR maternal OR postnatal OR postpartum OR pregnan*).tiab

Mental health terms

1. (mental health OR mental disorder OR mental illness* OR depress* OR anxi* OR stress*).tiab

Intervention terms

1. (intervention OR service OR treat* OR system OR innovati*) .tiab

Scaling or spreading  implementation terms

1. (spread OR spreading OR scale-up OR scaling OR scalability OR scal* up OR scal* out OR "bringing to scale" OR "bringing up to scale" OR "at scale" or "at capacity" OR diffus* OR expand*OR replicat* OR fidelity OR penetration OR uptake OR sustain* OR implement* OR adapt* OR adopt* OR disseminat*) .tiab

LMIC terms

1. (LMIC OR LAMIC OR low-middle-income OR low and  middle income OR low-resource OR resource poor) .tiab
2. (Afghanistan OR Albania OR Algeria OR "American Samoa" OR Angola OR Argentina OR Armenia OR Azerbaijan OR Bangladesh OR Belarus OR Belize OR Benin OR Bhutan OR Bolivia OR Bosnia And Herzegovina OR Botswana OR Brazil OR Bulgaria OR Burkina Faso OR Burundi OR "Cabo Verde" OR Cambodia OR Cameroon OR "Central African Republic" OR Chad OR China OR Colombia OR Comoros OR Congo OR Costa Rica OR "Cote D'ivoire" OR Cuba OR Djibouti OR Dominica OR "Dominican Republic" OR Ecuador OR Egypt OR "El Salvador" OR "Equatorial Guinea" OR Eritrea OR Eswatini OR Ethiopia OR Fiji OR Gabon OR Gambia OR Georgia OR Ghana OR Grenada OR Guatemala OR Guinea OR Guinea-Bissau OR Guyana OR Haiti OR Honduras OR India OR Indonesia OR Iran OR Iraq OR Jamaica OR Jordan OR Kazakhstan OR Kenya OR Kiribati OR Korea OR Kosovo OR Kyrgyz OR Lao OR Lebanon OR Lesotho OR Liberia OR Libya OR Madagascar OR Malawi OR Malaysia OR Maldives OR Mali OR "Marshall Islands" OR Mauritania OR Mauritius OR Mexico OR Micronesia OR Moldova OR Mongolia OR Montenegro OR Morocco OR Mozambique OR Myanmar OR Namibia OR Nepal OR Nicaragua OR Niger OR Nigeria OR Macedonia OR Pakistan OR Palau OR "Papua New Guinea" OR Paraguay OR Peru OR Philippines OR Russia OR Rwanda OR Samoa OR "Sao Tome And Principe" OR Senegal OR Serbia OR "Sierra Leone" OR "Solomon Islands" OR Somalia OR "South Africa" OR "South Sudan" OR "Sri Lanka" OR "St. Lucia" OR "St. Vincent and The Grenadines" OR Sudan OR Suriname OR Syria OR Tajikistan OR Tanzania OR Thailand OR Timor-Leste OR Togo OR Tonga OR Tunisia OR Turkiye OR Turkmenistan OR Tuvalu OR Uganda OR Ukraine OR Uzbekistan OR Vanuatu OR Vietnam OR West Bank OR Gaza OR Yemen OR Zambia OR Zimbabwe) .tiab
3. 5 OR 6
4. 1 AND 2 AND 3  AND 4  AND 7

**Supplementary Material 4. Extraction table**

| **ID** | **Database, Grey Literature or Citation?** | **Author** | **Year** | **Title** | **Link/ DOI** | **Paper type and design** | **Aim (copied from paper with page number)** | **Country** | **Context** | **Region** | **What is the intervention?** | **Process (Spread, Scale and/or Sustainability)** | **Final themes and subthemes** |
| --- | --- | --- | --- | --- | --- | --- | --- | --- | --- | --- | --- | --- | --- |
| 1 | Database | Agampodi et al. (2023) | 2023 | Incorporating early pregnancy mental health screening and management into routine maternal care: experience from the Rajarata Pregnancy Cohort (RaPCo), Sri Lanka | <https://doi.org/10.1136/bmjgh-2023-012852> | Journal Article: Practice paper | "to report the experience, our experience, observations and lessons learnt from a comprehensive maternal mental health screening programme, and the feasibility and utility of a health system that integrated simple interventions to improve maternal mental health." (pg 2) | Sri Lanka | Lower middle income | South Asia | Rajarata Pregnancy Cohort (RaPCo): A two-stage screening approach for antenatal anxiety and depression in early pregnancy and during the end of the second trimester, coupled with a package of personalised counselling, social and financial support and appropriate referral to the psychiatrist. | Scale | 1. Integration Primary care   2. Diversify workforce Non-specialist providers |
| 2 | Database | Ahmad et al. (2020) | 2020 | Measuring the implementation strength of a perinatal mental health intervention delivered by peer volunteers in rural Pakistan | <https://doi.org/10.1016/j.brat.2020.103559> | Journal Article: Development of an Implementation Strength Index embedded in a community-based cluster (RCT) | "to report the development of an Implementation Strength Index for a peer-delivered program for perinatal depression." (pg 2) | Pakistan | Lower middle income | South Asia | THPP: An adapted version of the Thinking Healthy Programme delivered by Peer Volunteers | Scale | 1. Tool and method development Implementation tools and methods |
| 3 | Database | Atif et al. (2016) | 2016 | Barefoot therapists: barriers and facilitators to delivering maternal mental health care through peer volunteers in Pakistan: a qualitative study | <https://doi.org/10.1186/s13033-016-0055-9> | Journal Article: Qualitative study | "to explore the facilitators and barriers to the acceptability of peer volunteers (PVs)—volunteer lay women from the community with shared socio-demographic and life experiences with the target population—as delivery agents of a psychosocial intervention for perinatal depression in a rural area of Pakistan." (pg 1) | Pakistan | Lower middle income | South Asia | THPP: An adapted version of the Thinking Healthy Programme delivered by Peer Volunteers | Scale | 1. Diversify workforce Peers |
| 4 | Database | Atif et al. (2017) | 2017 | Mother-to-mother therapy in India and Pakistan: adaptation and feasibility evaluation of the peer-delivered Thinking Health Programme | <https://doi.org/10.1186/s12888-017-1244-z> | Journal Article: Qualitative adaptation study | "to adapt the THP so that it could be delivered by peers to depressed mothers in the community in two different settings in India and Pakistan, and to test the adapted intervention for feasibility in a sample of peers and mothers in both settings." (pg 2) | Pakistan & India | Lower middle income | South Asia | THP: An evidence-based approach describing how community health workers can reduce perinatal depression through evidence-based cognitive-behavioural techniques | Spread Scale Sustainability | 1. Diversify workforce Peers   2. Adaptation Context (India)    3. Training, supervision, and support Train the trainer (cascade model) |
| 5 | Database | Atif et al. (2019a) | 2019 | Delivering maternal mental health through peer volunteers: a 5-year report | <https://doi.org/10.1186/s13033-019-0318-3> | Journal Article: Mixed methods evaluation | "[The THPP] has been iteratively adapted and continually delivered for 5 years in Pakistan. In this report, we describe the extended intervention  and factors contributing to the peers’ continued motivation and retention, and suggest future directions to address scale-up challenges." (pg 1) | Pakistan | Lower middle income | South Asia | THPP: An adapted version of the Thinking Healthy Programme delivered by Peer Volunteers | Scale  Sustainability | 1. Training, supervision, and support Train the trainer (cascade model)  2. Diversify workforce Peers |
| 6 | Database | Atif et al. (2019b) | 2019 | Scaling-up psychological interventions in resource-poor settings: training and supervising peer volunteers to deliver the ‘Thinking Healthy Programme’ for perinatal depression in rural Pakistan | <https://doi.org/10.1017/gmh.2019.4> | Journal Article: Mixed methods study as part of a RCT | "A cascaded model of training and supervision was developed to sustain delivery of an evidence-based peer-delivered intervention for perinatal depression (the Thinking Healthy Programme) in rural Pakistan. The study aimed to evaluate the model." (pg 1) | Pakistan | Lower middle income | South Asia | THPP: An adapted version of the Thinking Healthy Programme delivered by Peer Volunteers | Scale  Sustainability | 1. Diversify workforce Peers  2. Training, supervision, and support Train the trainer (cascade model) |
| 7 | Citation | Atif et al. (2020) | 2020 | Development of a psychological intervention to address anxiety during pregnancy in a low-income country | <https://doi.org/10.3389/fpsyt.2019.00927> | Journal Article: Qualitative adaptation study | "to develop a culturally appropriate, feasible, and acceptable psychological intervention for perinatal anxiety in the context of a low-income population in Pakistan." (pg 1) | Pakistan | Lower middle income | South Asia | THP: An evidence-based approach describing how community health workers can reduce perinatal depression through evidence-based cognitive-behavioural techniques | Spread | 1. Adaptation Condition (prenatal anxiety) |
| 8 | Database | Atif et al. (2022) | 2022 | Technology-assisted peer therapy: a new way of delivering evidence-based psychological interventions | <https://doi.org/10.1186/s12913-022-08233-6> | Journal Article: Co-design | "to develop a Technology-assisted peer delivered Thinking Healthy Programme for perinatal depression focusing on the needs of women living in resource-poor rural communities" (pg 2) | Pakistan | Lower middle income | South Asia | THPP: An adapted version of the Thinking Healthy Programme delivered by Peer Volunteers | Scale Sustainability | 1. Tool and method development Technology and digital solutions  2. Training, supervision, and support Techology and digital solutions   3. Diversify workforce Peers |
| 9 | Database | Atif et al. (2023) | 2023 | Non-specialist-delivered psychosocial intervention for prenatal anxiety in a tertiary care setting in Pakistan: a qualitative process evaluation | <https://doi.org/10.1136/bmjopen-2022-069988> | Journal Article: Qualitative process evaluation | 1. "to understand the factors impacting the intervention delivery process such as perceived benefits and acceptability as well as barriers and facilitators to successful intervention." (pg 2)  2. "to understand the research process such as challenges with participant recruitment and retention in a busy tertiary hospital and participants' views on the assessment procedures" (pg 2) - not included in extraction | Pakistan | Lower middle income | South Asia | Happy Mother Healthy Baby: An adapted version of the THP to target women with perinatal anxiety | Scale | 1. Diversify workforce Non-specialist providers  2. Integration Maternal and child health |
| 10 | Database | Bakare et al. (2017) | 2017 | Improving access to interventions among mothers screened positive for post-partum depression (PPD) at National Programme on Immunization (NPI) clinics in south-western and south-eastern Nigeria - a service development report | <https://doi.org/10.19185/matters.201707000005> | Journal Article: Service development report | "The study that was conducted in two phases of Piloting and Prototyping has the overall objective of improving access of mothers screened positive for postpartum depression to interventions and by that resolve their symptoms of depression, improve growth outcome in their infants and promote mother-child bonding." (pg 1) | Nigeria | Lower middle income | Sub-Saharan Africa | Service Innovation: Brought intervention services to primary healthcare centres at the National Programme on Immunization clinics | Scale | 1. Integration Primary care |
| 11 | Database | Bitew et al. (2022) | 2022 | Adapting an intervention of brief problem-solving therapy to improve the health of women with antenatal depressive symptoms in primary healthcare in rural Ethiopia | <https://doi.org/10.1186/s40814-022-01166-1> | Journal Article: Qualitative adaptation study | "to describe the process by which we selected and adapted a brief psychological intervention to  meet the needs of women experiencing antenatal depression in rural Ethiopia" (pg 2) | Ethiopia | Low income | Sub-Saharan Africa | MI-PST: Brief blended motivational interviewing and problem-solving therapy intervention delivered in the primary healthcare setting by peer counsellors | Spread | 1. Adaptation Context (Ethiopia) Condition (antenatal depressive symptoms) |
| 12 | Database | Boran et al. (2023a) | 2023 | Delivering the Thinking Healthy Programme as a universal group intervention integrated into routine antenatal care: a randomized-controlled pilot study | <https://doi.org/10.1186/s12888-022-04499-6> | Journal Article: Randomised controlled pilot study | "to pilot this adapted group intervention [THP-BGV] to evaluate its feasibility before a future definitive randomised controlled trial (RCT) and wider scale implementation." (pg 3) | Turkey | Upper middle income | Europe & Central Asia | THP-BGV: An online brief group version of the THP, designed to be integrated into the routine online antenatal pregnancy classes as a preventative intervention | Spread Scale | 1. Tool and method development Technology and digital solutions  2. Diversify workforce Non-specialist providers (nurses)  3. Integration Maternal and child health (antenatal education classes) Community models of care (preventative online course)  4. Adaptation Population (universal/preventative) |
| 13 | Citations | Boran et al. (2023b) | 2023 | Adaptation and integration of the Thinking Healthy Programme into pregnancy schools in Istanbul, Turkey | <https://doi.org/10.1186/s12884-023-05572-y> | Journal Article: Qualitative adaptation study | "to (a) adapt the THP reference manual-Turkish version for use in the group setting as a preventative intervention, (b) understand the relevance and acceptability of the adapted version to its delivery agents and expectant women, (c) explore its feasibility for integration into the existing antenatal care programme and, (d) explore online delivery." (pg 6) | Turkey | Upper middle income | Europe & Central Asia | THP: An evidence-based approach describing how community health workers can reduce perinatal depression through evidence-based cognitive-behavioural techniques | Spread Scale | 1. Adaptation Context (Turkey) Population (universal/preventative)  2. Integration Community models of care  3. Tool and method development Technology and digital solutions |
| 14 | Database | Davies et al. (2019) | 2019 | Psychotherapy for perinatal mental disorders in low-and middle-income countries | <https://doi.org/10.1016/B978-0-12-814932-4.00014-8> | Book Chapter | "We endorse a stepped-care model of mental health screening and service provision, integrated into public health services, using evidence-based psychotherapy for nonspecialist health-care workers with robust training and supervision." (abstract) |  | LMICs |  |  | Scale  Spread  Sustainability | 1. Integration Primary care  2. Diversify workforce  3. Stakeholder engagement *Participatory methods/Patient and public involvement  4. Adaptation |
| 15 | Database | Davies et al. (2022) | 2022 | Implementation of a task-shared psychosocial intervention for perinatal depression in South Africa: a grounded theory process evaluation | <https://doi.org/10.1016/j.ssmmh.2021.100056> | Journal Article: Qualitative process evaluation | "to conduct a process evaluation of a task-shared psychosocial intervention for perinatal depression in South Africa through a grounded theory analysis. More specifically, it aimed to identify processes that occurred within the delivery of counselling sessions in the intervention, and to use these findings to share recommendations for the development of future task-shared interventions for CMDs in LMICs." (pg 2) | South Africa | Upper middle income | Sub-Saharan Africa | AFFRIM-SA: A six-session task-shared manualised counselling intervention delivered by community health workers (CHWs) to depressed pregnant women at two Midwife and Obstetrics Units (MOUs). | Scale  Sustainability | 1. Diversify workforce Community health workers  2. Stakeholder engagement  3. Training, supervision, and support |
| 16 | Database | Fisher et al. (2014) | 2014 | Translation, cultural adaptation and field-testing of the Thinking Healthy Program for Vietnam | <https://doi.org/10.1186/1744-8603-10-37> | Journal Article: Qualitative adaptation study | "to examine whether the THP could be applied in universal programs to improve population-level perinatal mental health in Vietnam." (pg 3) | Vietnam | Lower middle income | East Asia & Pacific | THP: An evidence-based approach describing how community health workers can reduce perinatal depression through evidence-based cognitive-behavioural techniques | Spread | 1. Adaptation  Context (Vietnam) |
| 17 | Database | Green et al. (2020) | 2020 | Expanding access to perinatal depression treatment in Kenya through automated psychological support: development and usability study | <https://doi.org/10.2196/17895> | Journal Article: Mixed methods study | "to adapt Thinking Healthy for dissemination in Kenya through the Zuri AI system, develop and test study procedures to inform the design of a randomized controlled trial (RCT), and generate preliminary evidence of feasibility, acceptability, and response  to treatment." (pg 2) | Kenya | Lower middle income | Sub-Saharan Africa | Thinking Healthy (Healthy Moms): Adapting the THP to an existing artificial intelligence system called Tess (Zuri in Kenya). | Spread Scale | 1. Tool and method development Technology and digital solutions  2. Adaptation Context (Kenya) |
| 18 | Database | Honikman and Field (2020) | 2020 | Maternal mental health in South Africa and the opportunity for integration | <https://doi.org/10.1007/978-3-030-27080-3_27> | Book Chapter | Integration of mental health services into maternal care services | South Africa | Upper middle income | Sub-Saharan Africa | PMPH: An adaptable stepped-care, collaborative model for delivering mental health screening, counselling and case management | Scale Sustainability | 1. Integration  Maternal and child health  2. Training, supervision, and support  3. Stakeholder engagement |
| 19 | Database | Jabeen et al. (2023) | 2023 | Evidence of mobile health integration into primary health care systems for better maternal mental health in LMICs during COVID-19 pandemic - review | <https://doi.org/10.47391/jpma.5155> | Journal Article: Review | "to explore integration of maternal mental health into existing maternal, newborn and child health programs so that delivery of mental health interventions can be done alongside MNCH interventions." (pg 125) |  | LMICs |  |  | Scale | 1. Integration  Maternal and child health  2. Tool and method development Implementation tools and methods |
| 20 | Database | Keynejad et al. (2023) | 2023 | Adapting brief problem-solving therapy for pregnant women experiencing depressive symptoms and intimate partner violence in rural Ethiopia | <https://doi.org/10.1080/10503307.2023.2222899> | Journal Article: Qualitative adaptation study | "to adapt an evidence-based psychological intervention for pregnant women experiencing depressive symptoms and intimate partner violence in rural Ethiopia." (abstract) | Ethiopia | Low income | Sub-Saharan Africa | MI-PST: Brief blended motivational interviewing and problem-solving therapy intervention delivered in the primary healthcare setting by peer counsellors | Spread | 1. Adaptation Context (Ethiopia) Population (pregnant women) Condition (depression and IPV) |
| 21 | Database | Kohrt et al. (2022) | 2022 | The use of formative research to culturally adapt a psychosocial support program for perinatal Mayan women in Guatemala | <https://doi.org/10.1016/j.ssmmh.2022.100078> | Journal Article: Qualitative adaptation study | "to utilize a formative, mixed methods approach to 1) identify areas of the THP/PENSA program that require adaptation prior to implementation within a Tz'utujil Mayan community in Guatemala, and 2) develop a training protocol for local community health workers that is centred in their culture, context, and worldview." (pg 2) | Guatemala | Upper middle income | Latin America & Caribbean | THP: An evidence-based approach describing how community health workers can reduce perinatal depression through evidence-based cognitive-behavioural techniques | Spread | 1. Adaptation  Context (Guatemala) |
| 22 | Database | Le et al. (2023) | 2023 | Preventing perinatal depression: cultural adaptation of the Mothers and Babies Course in Kenya and Tanzania | <https://doi.org/10.3390/ijerph20196811> | Journal Article: Qualitative adaptation study | "to document how the Mothers and Babies Course (MBC), a cognitive–behavioural (CBT) intervention for perinatal mothers at risk for depression originally developed in the United States [Le et al., 2015], was adapted to fit the contexts of rural pregnant women and mothers of young children in Kenya and Tanzania using the FRAME." (pg 2) | Kenya & Tanzania | Lower middle income | Sub-Saharan Africa | Mothers and Babies Course (MBC): A cognitive–behavioural (CBT) intervention for perinatal mothers at risk of depression. | Scale  Spread | 1. Adaptation   2. Integration  Community models of care (Catholic Relief Services' Thrive II programme focused on early childhood development) |
| 23 | Database | Manolova et al. (2023) | 2023 | Integrating perinatal mental healthcare into maternal and perinatal services in low and middle income countries | <https://doi.org/10.1136/bmj-2022-073343> | Journal Article: Review | "argue for a comprehensive approach to the challenge of treating perinatal mental health conditions in maternal, neonatal, and child health services" (pg 1) |  | LMICs |  |  | Scale Sustainability | 1. Integration Maternal and child health   2. Diversify Workforce  3. Tool and method development Implementation tools and methods  4. Training, supervision, and support  5. Stakeholder engagement |
| 24 | Database | Marley et al. (2023) | 2023 | Maternal mental health in Sub-Saharan Africa: a systematic review of interventions for common perinatal mental health disorders | <https://doi.org/10.22541/au.168299829.99919269/v1> | Preprint: Review | "to collate the latest evidence and approaches to maternal mental health, and subsequent child outcomes, across low- and low-middle income countries in SSA, and to identify trends in practice to support practitioners and policy makers in programmatic design and delivery. Specifically, we will consider the characteristics of interventions to gain an understanding of what is being implemented, and where. Further, we will thematically analyse the adaptation process of interventions, looking at the dimensions of services and the form of service delivery (Castro et al., 2004) to better understand how they are implemented and to what extent this reflects the global guidance on management of CPMDs in low resource contexts." (pg 3) |  | LMICs | Sub-Saharan Africa |  | Spread Scale | 1. Adaptation  2. Diversify workforce |
| 25 | Grey Literature | McNab et al. (2021, developed by USAID's MOMENTUM) | 2021 | A landscape analysis: the silent burden: common perinatal mental disorders in low- and middle-income countries | <https://www.alignmnh.org/mmh-evidence/> | Journal Article: Review | "describes the current state of perinatal mental health and the burden of common perinatal mental disorders for women, newborns, and families in low- and middle-income countries (LMICs)." |  | LMICs |  |  | Scale Sustainability | 1. Integration   2. Diversify workforce  3. Tool and method development  Implementation tools and methods  4. Training, supervision, and support  5. Stakeholder engagement |
| 26 | Grey Literature | Mental Health Innovation Network | ND | Scale-up of a maternal depression intervention through technology in a post-conflict area | <https://www.mhinnovation.net/innovations/scale-maternal-depression-intervention-through-technology-post-conflict-area> | Website | "to scale up the Thinking Healthy Programme in a district affected by multiple humanitarian crises" | Pakistan | Lower middle income | South Asia | THP: An evidence-based approach describing how community health workers can reduce perinatal depression through evidence-based cognitive-behavioural techniques | Scale Sustainability | 1. Tool and method development Technology and digital solutions  2. Training, supervision, and support Train the trainer (cascade model) |
| 27 | Grey Literature | Mental Health Innovation Network | ND | Thinking Healthy pilot in Peru | <https://www.mhinnovation.net/innovations/thinking-healthy-pilot-peru?mode=wyocyiyvuws> | Website | "to reduce maternal depression and strengthen emotional skills in mothers through a community-based platform of care" | Peru | Upper middle income | Latin America & Caribbean | THP: An evidence-based approach describing how community health workers can reduce perinatal depression through evidence-based cognitive-behavioural techniques | Scale  Spread  Sustainability | 1. Adaptation  Context  2. Diversify workforce Community health workers  3. Training, supervision, and support  4. Stakeholder engagement |
| 28 | Database | Nisar et al. (2020) | 2020 | Making therapies culturally relevant: translation, cultural adaptation and field-testing of the Thinking Healthy Programme for perinatal depression in China | <https://doi.org/10.1186/s12884-020-03044-1> | Journal Article: Qualitative adaptation study | "to translate and adapt Thinking Health Programme (THP) for the Chinese population and to establish its acceptability when delivered by non-specialists to a group of mothers with perinatal depression." (pg 2) | China | Upper middle income | East Asia & Pacific | THP: An evidence-based approach describing how community health workers can reduce perinatal depression through evidence-based cognitive-behavioural techniques | Spread | 1. Adaptation |
| 29 | Database | Obonyo et al. (2023) | 2023 | Diverse policy maker perspectives on the mental health of pregnant and parenting adolescent girls in Kenya: considerations for comprehensive, adolescent-centered policies and programs | <https://doi.org/10.1371/journal.pgph.0000722> | Journal Article: Qualitative study | "examines the perspectives of diverse policy makers on mental health promotion and prevention among pregnant and parenting adolescent girls to help identify points of entry and barriers to developing policies, programs, and messaging for mental health promotion among peripartum adolescent populations." (pg 3) | Kenya | Lower middle income | Sub-Saharan Africa |  | Sustainability | 1. Stakeholder engagement |
| 30 | Database | Owais et al. (2023) | 2023 | Integrating maternal depression care at primary private clinics in low-income settings in Pakistan: a secondary analysis | <https://doi.org/10.3389/fgwh.2023.1091485> | Journal Article: Mixed methods study | "to assess the implementation process of the maternal depression intervention component to gain insight into the experiences, feasibility, and fidelity of delivering a counselling package at primary care clinics in low-resource settings." (pg 2) | Pakistan | Lower middle income | South Asia | Maternal depression intervention: A secondary component of an early child development (ECD) randomized control trial focusing on infants aged 0–12 months. | Scale Sustainability | 1. Integration  Primary care   2. Stakeholder engagement |
| 31 | Grey Literature | The Partnership for Maternal, Newborn, & Child Health | 2014 | Maternal mental health: why it matters and what countries with limited resources can do | <https://www.gammh.org/> | Report: Knowledge summary | Report on why maternal mental health matters and what countries with limited resources can do |  | LMICs |  |  | Scale Sustainability | 1. Integration  Maternal and child health  2. Stakeholder engagement |
| 32 | Grey Literature | Perinatal Mental Health Project | ND | Perinatal Mental Health Project: to address the treatment gap for perinatal mental disorders in South Africa | <https://pmhp.za.org/> | Website | "to support the integration of quality maternal mental health care into maternal and child platforms to optimise access to health, development and to social justice." | South Africa | Upper middle income | Sub-Saharan Africa | PMPH: An adaptable stepped-care, collaborative model for delivering mental health screening, counselling and case management | Scale Sustainability | 1. Diversify workforce Non-specialist providers  2. Integration Maternal and child health  3. Stakeholder engagement   4. Training, supervision, and support |
| 33 | Grey Literature | Prime (UK Aid ID, 2016) | 2016 | PRIME (programme for improving mental health care): evidence on scaling-up mental health services for development | <https://assets.publishing.service.gov.uk/media/57a089b340f0b64974000208/PRIMEbrochure.pdf> | Report: Programme brochure | "to generate high-quality research evidence on the implementation and scaling up of treatment programmes for priority mental disorders in primary healthcare contexts in 5 low resource country settings." | Ethiopia, India, Nepal, South Africa & Uganda | LMICs | Sub-Saharan Africa/South Asia |  | Scale Sustainability | 1. Integration  Primary care  Maternal and child health  2. Stakeholder engagement |
| 34 | Database | Sarkar et al. (2020) | 2020 | Integration of perinatal mental health care into district health services in Uganda: why is it not happening? The Four Domain Integrated Health (4DIH) explanatory framework | <https://doi.org/10.1016/j.socscimed.2020.113464> | Journal Article: Qualitative study | "aimed at qualitatively situating the extent to which integration of perinatal mental health care into maternal mental health care was considered desirable, possible and opportunities within the existing policy and service-delivery environment in Uganda." (abstract) | Uganda | Low income | Sub-Saharan Africa |  | Scale Sustainability | 1. Tool and method development Implementation tools and methods  2. Integration  Community health workers  3. Stakeholder engagement |
| 35 | Database | Singla et al. (2014) | 2014 | “Someone like us”: delivering maternal mental health through peers in two South Asian contexts | <https://doi.org/10.1016/j.jad.2014.07.017> | Journal Article: Qualitative study | "to address key questions on the characteristics of human resources outside the formal health care system to deliver THP to mothers with depression." (pg 453) | Pakistan & India | Lower middle income | South Asia | THPP: An adapted version of the Thinking Healthy Programme delivered by Peer Volunteers | Scale | 1. Diversify workforce  Peers |
| 36 | Database | Suchman et al. (2020) | 2020 | Mothering from the inside out: adapting an evidence-based intervention for high-risk mothers in the Western Cape of South Africa | <https://doi.org/10.1017/S0954579418001451> | Journal Article: Mixed methods study | "to establish a long-term collaborative relationship based on CBPR principles between members of the research team that developed and evaluated MIO in the U.S. and psychosocial treatment providers in four public sector (tertiary) university-affiliated hospitals in the Western Cape. The primary objective of the collaboration was to adapt MIO for delivery with mothers caring for children in extremely stressful environments where mothers and/or children’s mental or physical health had been compromised." (pg 8) | South Africa | Upper middle income | Sub-Saharan Africa | Mothering from the Inside Out (MIO): A mentalization-based intervention for mothers. | Spread | 1. Adaptation Context |
| 37 | Grey Literature | UNFPA/ WHO inter-national expert meeting (2009) | 2009 | Maternal mental health and child health and development in resource-constrained settings | <https://www.who.int/publications/i/item/WHO-RHR-09.24> | Report: International meeting report | "The meeting brought together the world’s leading researchers in this field. They sought to assess the status of knowledge concerning the perinatal mental health problems of women in resource- constrained settings, their effects on infants, and the effectiveness of low-cost interventions." (pg 1) |  | LMICs |  |  | Scale  Sustainability | 1. Integration Primary care  Maternal and child health  2. Stakeholder engagement |
| 38 | Database | Waqas and Rahman (2023) | 2023 | Innovations in scaling up interventions in low-and middle-income countries: parent-focused interventions in the perinatal period and promotion of child development | <https://doi.org/10.1016/B978-0-323-91709-4.00010-X> | Book Chapter | "Given the high burden and global health relevance of perinatal anxiety and depression, this chapter is largely focused on these two conditions." (pg 257)  "Before discussing available microlevel interventions in LMICs, we examine the issue of screening of individuals who may be in need of such interventions. We use the example of perinatal common mental disorders to discuss the challenges involved in mass population screening, especially in low-resource settings." (pg 258) |  | LMICs |  |  | Scale Sustainability | 1. Diversify workforce   2. Integration  Maternal and child health   3. Tool and method development Technology and digital solutions  Implementation tools and methods   4. Training, supervision, and support  Train the trainer (cascade model) Technology and digital solutions |
| 39 | Database | Waqas et al. (2022) | 2022 | Predicting remission among perinatal women with depression in rural Pakistan: a prognostic model for task-shared interventions in primary care settings | <https://doi.org/10.3390/jpm12071046> | Journal Article: Presentation of model | "to develop and validate an easy-to-implement clinical prediction tool to assess prognosis and treatment response in task-shared intervention programs in primary care settings." (pg 3) | Pakistan | Lower middle income | South Asia | THP: An evidence-based approach describing how community health workers can reduce perinatal depression through evidence-based cognitive-behavioural techniques | Scale | 1. Tool and method development Technology and digital solutions |
| 40 | Grey Literature | WHO (2022b) | 2022 | Guide for integration of perinatal mental health in maternal and child health services | <https://www.who.int/publications/i/item/9789240057142> | Report: WHO guide | "to provide information about how staff in MCH services can provide mental health promotion, prevention, treatment and care." (pg 2) "to be used to develop and sustain high-quality, integrated mental health services for women during the perinatal period." (pg 2) |  | LMICs |  |  | Scale  Spread Sustainability | 1. Integration  Maternal and child health   2. Diversify workforce   3. Training, supervision, and support   4. Adaptation |
| 41 | Database | Zafar et al. (2014) | 2014 | Integrating maternal psychosocial well-being into a child-development intervention: the five-pillars approach | <https://doi.org/10.1111/nyas.12339> | Journal Article: Presentation of model | "In this paper, we describe the development and piloting of an approach for MPW that has the potential for integration at scale in a combined nutrition and early child development program. The approach is designed to be delivered by a CHW as part of any maternal or child health program." (pg 108) | Pakistan | Lower middle income | South Asia | THP: An evidence-based approach describing how community health workers can reduce perinatal depression through evidence-based cognitive-behavioural techniques | Scale  Spread | 1. Adaptation Condition (universal MPW)  2. Integration  Maternal and child health (nutrition programme)  3. Diversify workforce  Community health workers |
| 42 | Database | Zhu et al. (2022) | 2022 | Factors affecting the implementation of task-sharing interventions for perinatal depression in low- and middle-income countries: a systematic review and qualitative metasynthesis | <https://doi.org/10.1016/j.jad.2022.01.005> | Journal Article: Review | "to use the CFIR to identify factors critical to the successful implementation of PND task-sharing interventions in LMICs and to weigh their levels of evidence." (pg 401) |  | LMICs |  |  | Scale | 1. Diversify workforce |
